# Supplementary material for: Estimating relative risk of within-lake aquatic plant invasion using combined measures of recreational boater movement and habitat suitability
Source: PeerJ. 2015 Mar 19;3:e845. doi: 10.7717/peerj.845 (PMC4369337; doi:10.7717/peerj.845)
Supplement: Supplemental Information 1 — Questionnaire administered to recreational boaters concerning movements within Lake Tahoe. Question #’s 3, 4, 5 were used in this study. [file peerj-03-845-s001.pdf]

Supplement S1. Lake Tahoe Boater Survey Form

Launch________________ Boat type_____________ Time ________ Date___________

1) Where are you from (ZIP)? Number of trips to Tahoe per year

2) Where was last use of this boat and when? Where is your next planned use of this boat and when?

3) Have you visited any other marinas, harbors, launches during your time today? (Ordered visits)

4) While there, did you stop your boat? Anchor it?

5) While on the lake, have you boated through any aquatic plants today? EWMF?

6) Where do you usually store your boat? Parked outside, buoyed, slip?

7) Before you transported the boat(s) from Lake Tahoe, did you notice any aquatic plant fragments, such as Eurasian watermilfoil stuck on your boat(s) propellers or trailers?

8) Have you taken any steps to remove plant fragments from your boat/trailer upon leaving the lake?

9) After removing boat(s) from the water, how often do you do the following? How do you clean your boat?

	Steps taken:	Almost Always	Some-times	Never	Does not Apply
a.	Conduct visual inspection of boats and equipment for aquatic plants	1	2	3	4
b.	Remove aquatic plants from boats and equipment	1	2	3	4
c.	Rinse boat with high pressure and/or hot water	1	2	3	4
d.	Allow boat to dry for at least five days	1	2	3	4
e.	Other (please specify) ____	1	2	3	4

10) Have aquatic plant species caused problems for you or affected your recreational experience today or at other times during the 2005 boating season?

Inspections:

Plants on props, outboards, trailer, etc.? Yes No

ID: ____

Time Collected: _________________
